# Pain Reduction during Rapid Palatal Expansion Due to LED Photobiomodulation Irradiation: A Randomized Clinical Trial

**DOI:** 10.3390/life12010037

**Published:** 2021-12-27

**Authors:** Gianluigi Caccianiga, Paolo Caccianiga, Marco Baldoni, Antonino Lo Giudice, Letizia Perillo, Nicolò Moretti, Saverio Ceraulo

**Affiliations:** 1 School of Medicine and Surgery, University of Milano-Bicocca, 20900 Monza, Italy; gianluigi.caccianiga@unimib.it (G.C.); marco.baldoni@unimib.it (M.B.); saverio.ceraulo@unimib.it (S.C.); 2 Department of General Surgery and Surgical-Medical Specialties, Unit of Orthodontics, School of Dentistry, University of Catania, Via S. Sofia 78, 95124 Catania, Italy; nino.logiudice@gmail.com; 3 School of Medicine and Surgery, University of Campania Luigi Vanvitelli, 80138 Naples, Italy; letizia.perillo@unicampania.it; 4 Private Practitioner, 22012 Cernobbio, Italy; n.moretti@campus.unimib.it

**Keywords:** photobiomodulation, LED, orthodontic, pain

## Abstract

*Objective:* The purpose of this research is to assess the analgesic efficiency of Photobiomodulation (PBM) in pain reduction in young patients during rapid maxillary expansion therapy. *Materials and Methods*: Thirty patients were included and allocated to an experimental group [Rapid Palatal Expansion (RPE) and PBM] and a control group (RPE only) at random. Inclusion criteria were skeletal age from CVS1 to CVS3, examined on the cephalometric lateral teleradiography, with cervical vertebra staging and completed eruption of upper first molar. Exclusion criteria were previous orthodontic treatment, bone disease, disability, or skeletal age from CVS4. Patients referred the pain they felt using a Numerical scale rate (NRS), ranging from 0 to 10, with specific intervals of time: 6 h, 12 h, 24 h, and from days 2 to 7. The Wilcoxon-Mann-Whitney test was used to evaluate differences in NRS reported values between the two groups. *Results:* The final sample included 30 patients, 14 males and 16 females, with a mean age of 7.8 ± 1.2 years. The pain that was felt at each time interval and the maximum score of pain were significantly lower in the experimental group (*p* < 0.05) and decreased faster in the experimental group, with a score test near to 0 after 2/3 days. *Conclusions*: PBM is efficient in reducing the intensity and the time of pain felt by young patients that undergo rapid maxillary expansion.

## 1. Introduction

Rapid maxillary expansion (RME) is the treatment of choice for the correction of transversal maxillary deficiency and posterior cross-bite in growing subjects [1]. This treatment is generally performed using a tooth-anchored maxillary expander device [2]. In this regard, RME generates heavy forces that are transferred to the mid-palatal suture, by the way of the teeth, thus leading to skeletal expansion [3,4,5]. When this force exceeds the resistance of the maxillary sutural articulations, the maxillary palatal suture separates, and skeletal expansion begins [6]. However, undesired effects in the maxillary posterior dento-alveolar structures have been reported, such as dental tipping [2,7], pulp modification [8,9], root resorption [10], and buccal bone loss [11]. Moreover, the effects of RME are not limited to the maxillary alveolus and midpalatal suture but are expected to affect several other adjacent structures in the face and the cranium [12,13,14].

Concerning patients’ experience, previous evidence would suggest that pain is the symptom mostly reported, with a frequency >90% among children [15]. Orthodontic pain has a multifactorial etiology and can be influenced by different factors such as gender, age, pain threshold, stress, race, as well as the magnitude of force applied. During RME protocol, the inflammatory process occurring during sutural opening and the compression of the periodontal ligament, could significantly contribute to the pain experienced [16].

Photobiomodulation is a therapy with many different applications in medicine and dentistry and it is free of side effects [17,18]. It consists in the usage of low-powered laser light within the red to near-infrared range (wavelengths from 632 to 1064 nm) in order to obtain biological responses. PBM seems to be able to reduce orthodontic pain in two ways [19,20]: it inhibits the release of arachidonic acid, which decreases the levels of prostaglandin E, and it provokes the release of beta-endorphins, which induce an efficient analgesic reaction. Meanwhile, recent evidence from randomized trials confirmed that PBM can be used to reduce orthodontic acute pain [21,22,23,24]; however, the literature still lacks studies assessing the same effect during the administration of heavy orthodontic forces, such as those produced for facial orthopedic treatment. In this regard, the purpose of the present clinical trial was to demonstrate the effect of PBM on the pain witnessed after rapid palatal expansion in a prospectively recruited cohort of growing subjects.

## 2. Materials and Methods

The present randomized clinical trial, with single operator and parallel groups (1:1) was performed with respect to the Declaration of Helsinki of 2013. This study does not have a clinical trial registration number, because it is a strictly observational study: we simply reported the pain perceived by patients undergoing rapid palatal expansion treatment, some who had a Photobiomodulation session with ATP38 (an absolutely noninvasive or harmful device, risk-free, and beneficial in many respects, thanks to its analgesic and anti-inflammatory properties), and some who did not. We did not measure the pain with any device, we limited ourselves by pain reported by patients.

Patients were chosen and treated in the period between April 2019 and March 2020, and they all signed an accurate informed consent.

Patients were chosen following these criteria: (1) skeletal age from CVS1 to CVS3, determined with the cervical vertebra staging on the cephalometric lateral teleradiography, (2) complete eruption of the first upper molars, and (3) clinical need to undergo an RPE. Some patients were excluded following these other criteria: (1) skeletal age from CVS4, (2) craniofacial disorders or bone diseases, (3) orthodontic treatment in the past, and (4) disability.

Patients were divided at random into a group that received RPE and LED irradiation and group that received RPE only following a randomized balanced block protocol that had sex and ages as stratification factors.

Patients belonging to the control group were treated without LED irradiation at the Odontostomatology department of the San Gerardo Hospital of Monza (University of Milan-Bicocca). As for the patients belonging to the experimental group, they were treated with LED irradiation in a private clinic in the province of Bergamo.

For randomization purposes, we used the SPSS Statistics software (IBM Corporation, Armonk, NY, USA) to generate an allocation sequence.

A total of 151 patients were recruited from a larger sample of patients, 117 of whom were excluded because the inclusion criteria were not met.

**Figure 1:**
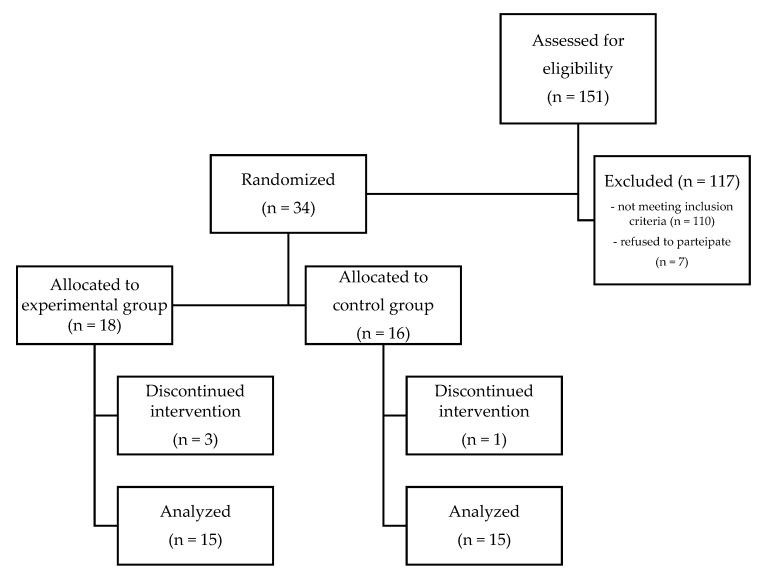
CONSORT flowchart.

Of the remaining 34 patients, 18 were assigned to the experimental group and 16 to the control group (Table 1; Figure 1). This difference in the number of the two groups is due to the fact that we wanted to have a greater number of patients treated with LED radiation, to have more results in case of the further exclusion of patients. In fact, 3 patients were excluded from the research, because they did not correctly complete or submit the questionnaire with the NRS scale for each time report, and one patient was excluded from the study for taking anti-inflammatory drugs. In the end, we were able to analyze 15 patients from the experimental group and 15 from the control group.

**Figure 2:**
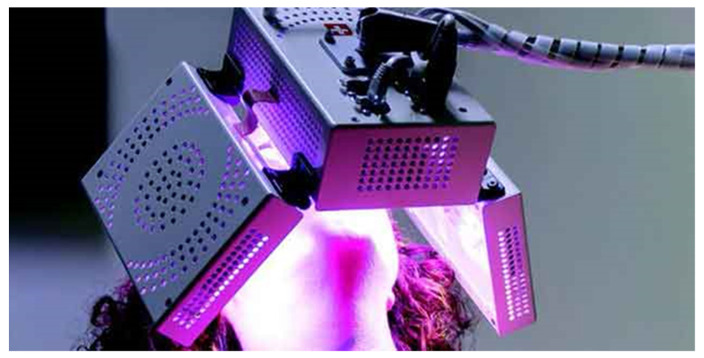
Extraoral LED appliance with ATP38^®^.

We took the alginate impressions for the execution of the rapid expansion device with bands on the first permanent molars and we cemented them with glass ionomer cement (3M ™ Ketac ™ Cem). We used the same protocol for the palatal expansion for both the test and control groups, 2 activations per day were executed distant 12 h from each other for an overall period of 7 days (2/4 turn per day, 0, 5 mm per day). No LED biostimulation was performed on the control group. The test group, on the other hand, was treated with LED irradiation at the time of the positioning of the RPE with ATP38^®^ (Biotech Dental, Allée de Craponne, Salon De Provence, France) (Figure 2). This device consists of a multi-plate system emitting cold polychromatic light with a combination of wavelength from 450 to 835 nm. For the aims of this research, the photobiomodulation scheme used, in line with the producer’s indications, involves 6 min of irradiation obtaining 48 J/cm^2^ of fluence, measured as the summation of the fluences created by the light source (16 J/cm^2^) of each of the three active panels (16 J/cm^2^ × 3 = 48 J/cm^2^). These standards are based on a specified distance of 4 cm of the side panels from the cheeks and of the side panel from the lips. We used 3 consecutives cycles of PBM, because 48 J/cm^2^ are lower than the fluence range used for orthodontic PBM, for an overall time of 18 min and 144 J/cm^2^ of fluence (48 J/cm^2^ × 3 cycles) with 1 min suspension between each cycle. The level of pain perceived by each participant was assessed with the NRS (numerical rating scale) at a distance of 6, 12, 24 h, and after each day up to the seventh day from the positioning of the expander in situ. Verbal instructions were provided to parents and young patients on how to correctly evaluate the perceived pain level, to indicate on the scale a value from 0 or absence of pain to 10 or the maximum pain imaginable. No one of those analyzed used analgesics or anti-inflammatory drugs during the active expansion phase. The collection, processing, and storage of data took place through electronic questionnaires, which can be filled in online and were accessible to each participating researcher. The data were recorded electronically and analyzed.

### Statistical Analysis

For both test and control groups, gender distribution was analyzed, descriptive statistics, mean, and standard deviation of age were calculated, and data reported on the NRS scale for all time intervals analyzed by T0 (day of positioning of the device in situ) at time T1 (at the end of the first week of activations), with the use of Excel worktables. Using the Shapiro-Wilk normality test (Social Science Statistic, 2019), the normal distribution of the data obtained was preliminarily checked (Shapiro and Wilk, 1965). Since the data were not normally distributed, a nonparametric test was chosen to evaluate the statistical significance of the analyzed values. The differences in the NRS assess between the experimental and control group at each time record were analyzed by the Wilcoxon-Mann-Whitney nonparametric statistical test (Motulsky, 2007). In addition, a linear regression was used to evaluate the effect of timing on pain experienced within the entire period. Statistical significance was set as *p* ≤ 0.05.

## 3. Results

The statistical analysis of the data obtained from the questionnaires completed by the patients of the control group and the experimental group, irradiated according to the ATP38^®^ laser protocol, showed the following results.

**Figure 3:**
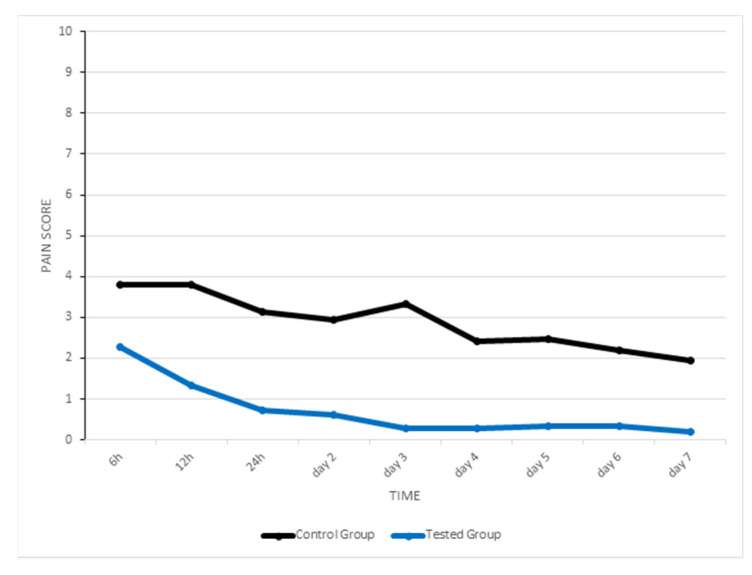
A graphic which represents change in pain intensity over time in test and control group.

In the test group, the highest perception of pain was at six hours after the expander was placed, with an intermediate scoring of 2, and then it progressively decreased to an intermediate scoring of 1 after 12 h, and 0 from day two (Table 2; Figure 3).

In the control group, the highest perception of pain was at 6 and 12 h after the expander was placed, with an intermediate scoring of 4, and then it initially declined to a median of 3 from 24 h, but then increased again at day three to a median of 4, decreasing to 2 on day four, 3 on day five, 2 on day six, and 1 on day seven (Table 2; Figure 3).

The Mann-Whitney showed that the pain perceived by patients in each moment we recorded and the highest pain scored were significantly different between the two groups (Table 2). In this regard, the scores were considerably lower in the experimental group.

A strong correlation was found between the timing (assessed as multiple follow-up) and pain perceived in control group (R = 0.933) and the experimental group (R = 0.750), according to the linear regression model. The R-squared value, which reflects the percentage of variability in each dependent variable (pain) attributed to the changes of the predictor (time), was 0.852 in the control group and 0.563 in the experimental group (Table 3).

## 4. Discussion

The purpose of this randomized clinical trial was to assess the effects of LED photobiomodulation on pain perception during rapid expansion of the maxilla in growing subjects. Previous studies have analyzed the trend of perceived pain intensity during rapid expansion of the palate showing a peak in the first three days due to the release of the chemical mediators of inflammation and the creation of hyalinization zones, following the application of orthodontic strength. Our results are consistent and confirm this pattern. First of all, we observed that in the literature there were studies that analyzed the effectiveness of laser photobiomodulation in orthodontics during the positioning of elastic separators [25,26] or orthodontic archwires and evaluate their analgesic efficacy according to the protocols [27,28]. There were several works on the efficacy of lasers and LEDs, not only in orthodontics but even in other fields of dentistry, especially in the management of periodontitis and perimplantitis [29,30,31,32,33,34,35]. However, no study has ever involved the analgesic efficacy of the laser in palatal expansion [36].

Certainly, a first reason lies in the fact that the application of a correct biostimulation must face the technical difficulties inherent in the type of device used. In fact, ATP38^®^ allows a simultaneous irradiation of all circummaxillary sutures in clinically acceptable times, difficult to achieve with other types of laser machines managed directly by the operator. The operator dependency parameter, which can certainly represent a bias for the statistical evaluation and a reproducibility constraint of the study itself, was eliminated by the standardization of the irradiation protocol [37]. Animal studies have shown that Photobiomodulation can reduce the inflammatory process in a comparable way to the use of NSAIDs and anti-inflammatories. In addition, PBM can improve blood supply and speed up tissue healing, playing a fundamental role in improving the healing of both hard and soft tissues. Other determining factors that contribute to the analgesic effect are the reactivation of enzymes targeted by the factors produced during the nociceptive response, the inhibition of depolarization of the C fibers, the production of ATP, and the reduction in prostaglandins. Finally, nerve conduction is also altered thanks to the synthesis, release, and metabolism of endorphins and encephalins and other important neuromediators. The rationale behind the choice of the ATP38^®^ device was dictated by the analysis of the biological effects on the tissues, by the analgesic efficacy shown by the LEDs in the literature, but above all by the possibility of eliminating the operator-related bias, with a saving of time useful for the clinician, and the ability to control the setting and parameterization of the instrument itself with extreme precision and reproducibility [38,39,40,41]. The limits of the present study were dictated by the size of the sample analyzed, which, however, was not marred by the heterogeneity of the sample itself, including a pool of patients homogeneous in terms of age and gender distribution, although clinical evidence is absent in the literature about the differences in the algic response and the pain sensitivity between males and females. Another limitation of the study can be considered the subjective nature of the assessments that the individual patients gave in first person of the pain and the absence of a placebo group to analyze the interference of the patient’s expectation on the perception of the pain itself.

According to our statistics, the pain perceived by subjects who endured LED appliance was significantly lower than that experienced in control group. Similarly, the maximum pain score was significantly lower. Based on the study of Farrar et al. [42], a decrease of around 2 points in the NRS describes a standard of a clinically considerable change. The differences observed in our data were equivalent or above 2 points confirming the clinical significance of our findings. According to our outcomes we would suggest the utilization of ATP38^®^ laser for reducing orthodontic pain related to RPE for these reasons: (1) demonstrated analgesic effect with no known undesirable effects on tissues, (2) the secondary effects reported with the use of analgesic drugs, and (3) the benefits of an operator-free LED appliance in gaining of time and precision of the device setting provided.

## 5. Conclusions

LED photobiomodulation with ATP38^®^ laser alleviates the intensity and duration of pain perceived by few patients enduring rapid maxillary expansion.

It follows that the use of LED irradiation with ATP38^®^ could be an important aid for the orthodontist professional in the treatment of young patients with rapid expansion of the palate, as the reduction in the intensity and duration of pain would certainly benefit the patient and their compliance would increase, as well as the parents’ willingness to continue with further orthopedic-orthodontic therapies necessary for a harmonious growth of the dento-facial complex.

## Figures and Tables

**Table 1:** Demographic and descriptive statistics of the study sample. (EG: experimental group; CG: control group).

Sample Characteristics	Total Sample	EG (n = 15)	CG (n = 15)	Significance
Gender: male/female	14/16	7/8	7/8	NS
Age, y, mean (SD)	7.8 (1.2)	7.6 (1.1)	8 (1.2)	NS

**Table 2:** Maximum pain score and pain experienced at each time schedule, assessed via the Numeric Rating Scale EG, experimental group, CG control group, 95% IC, 95% confidence interval. *p* value set as ≤0.05 and assessed by Mann-Whitney test.

Time Schedule	Median CG (min-max) IC 95%	Median EG (min-max) IC 95%	Significance
6 h	4 (1–7), IC 95%: 2.75–4.85	2 (1–4), IC 95%: 1.73–2.80	<0.05
12 h	3 (2–7), IC 95%: 2.93–4.67	1 (0–3), IC 95%: 0.84–1.83	<0.001
24 h	3 (1–6), IC 95%: 2.30–3.97	1 (0–2), IC 95%: 0.29–1.18	<0.001
2 d	3 (0–5), IC 95%: 2.03–3.83	0 (0–2), IC 95%: 0.19–1.01	<0.001
3 d	4 (1–7), IC 95%: 2.45–4.21	0 (0–1), IC 95%: 0.01–0.52	<0.001
4 d	2 (0–5), IC 95%: 1.68–3.12	0 (0–1), IC 95%: 0.01–0.52	<0.001
5 d	3 (0–6), IC 95%: 1.53–3.40	0 (0–1), IC 95%: 0.06–0.60	<0.001
6 d	2 (0–6), IC 95%: 1.24–3.16	0 (0–1), IC 95%: 0.06–0.60	<0.05
7 d	1 (0–6), IC 95%: 0.88–2.99	0 (0–1), IC 95%: 0.03–043	<0.05

**Table 3:** Linear regression model using time as the independent variable (predictor) and pain as the dependent variable assessed in the control group (CG) and experimental group (TG).

Dependent Variable	Predictor Variable	R	R-Square	Standard Error	Significance
Pain CG	Time	0.933	0.852	0.262	*p* < 0.001
Pain TG	Time	0.750	0.563	0.485	*p* < 0.05

## Data Availability

The data supporting the findings of the article are available from the corresponding author upon request.
